# Bedaquiline-based treatment for extensively drug-resistant tuberculosis in South Africa: A cost-effectiveness analysis

**DOI:** 10.1371/journal.pone.0272770

**Published:** 2022-08-05

**Authors:** Ginenus Fekadu, Jiaqi Yao, Joyce H. S. You

**Affiliations:** Faculty of Medicine, School of Pharmacy, The Chinese University of Hong Kong, Hong Kong, SAR, China; Murdoch University, AUSTRALIA

## Abstract

**Background:**

The treatment success rate of conventional anti-tuberculosis (TB) regimens for extensively drug-resistant TB (XDR-TB) is low, resulting in high morbidity and healthcare cost especially in the high TB burden countries. Recent clinical findings reported improved treatment outcomes of XDR-TB with the bedaquiline (BDQ)-based regimens. We aimed to evaluate the cost-effectiveness of BDQ-based treatment for XDR-TB from the perspective of the South Africa national healthcare provider.

**Methods:**

A 2-year decision-analytic model was designed to evaluate the clinical and economic outcomes of a hypothetical cohort of adult XDR-TB patients with (1) BDQ-based regimen and (2) injectable-based conventional regimen. The model inputs were retrieved from literature and public data. Base-case analysis and sensitivity analysis were performed. The primary model outputs included TB-related direct medical cost and disability-adjusted life years (DALYs).

**Results:**

In the base-case analysis, the BDQ group reduced 4.4152 DALYs with an incremental cost of USD1,606 when compared to the conventional group. The incremental cost per DALY averted (ICER) by the BDQ group was 364 USD/DALY averted. No influential factor was identified in the sensitivity analysis. In probabilistic sensitivity analysis, the BDQ group was accepted as cost-effective in 97.82% of the 10,000 simulations at a willingness-to-pay threshold of 5,656 USD/DALY averted (1× gross domestic product per capita in South Africa).

**Conclusion:**

The BDQ-based therapy appeared to be cost-effective and showed a high probability to be accepted as the preferred cost-effective option for active XDR-TB treatment.

## Introduction

Extensively drug-resistant tuberculosis (XDR-TB) is a highly drug-resistant infection, and 6.2% of multidrug-resistant TB cases were XDR-TB [1]. XDR-TB is caused by *Mycobacterium tuberculosis* strains which are resistant to isoniazid, rifampin, any fluoroquinolone, plus either bedaquiline or linezolid [1]. XDR-TB poses a global public health emergency due to limited therapeutic options, prolonged treatment duration, and high health economic burden [2,3]. The World Health Organization (WHO) reported over 25,000 laboratory-confirmed pre-XDR-TB or XDR-TB cases globally in 2020 [1].

South Africa is a country with high burden of XDR-TB [1,4]. The management cost per case of XDR-TB was USD26,392 in this endemic setting, higher than those of multidrug-resistant TB (USD6,772) and drug-sensitive TB (USD257) by 4-fold and 100-fold, respectively [5]. The conventional injectable (capreomycin)-based anti-TB regimens for XDR-TB are associated with low treatment success rate (10.3%-20%) [6–8]. The conventional treatment is also characterized by a high adverse event rate (58%) [9].

Bedaquiline (BDQ) is a novel oral drug indicated for the treatment of drug-resistant tuberculosis [10]. Recent clinical trials and large epidemiological cohort studies reported high sputum culture conversion (54.1%-75%) among XDR-TB patients treated by BDQ-based anti-TB regimens [7,11,12]. The BDQ-based regimens are also reported to be better tolerated than conventional therapy. A large cohort analysis in China (n = 1162 received BDQ-based regimens) reported that 66.9% of the adverse events were classified as minor events (grade 1–2) [13]. QT-prolongation was reported infrequently (0.4%) [12], and gradually declined after BDQ treatment was discontinued [14].

Replacing conventional injectable-based regimen with BDQ-based therapy has been showed to improve effectiveness and safety of active XDR-TB treatment. A recent systematic review of pharmacoeconomic evalutions of BDQ-based treatment yet found no reported cost-effectiveness analysis for treatment of active XDR-TB in high burden countries [15]. To faciliate the informed decision-making process on public healthcare resource allocation for XDR-TB treatment in high burden countries, this study aimed to evaluate the cost-effectiveness of BDQ-based regimens for the treatment of XDR-TB from the perspective of the South Africa national health service provider.

## Methods

### Model structure

**Fig 1:**
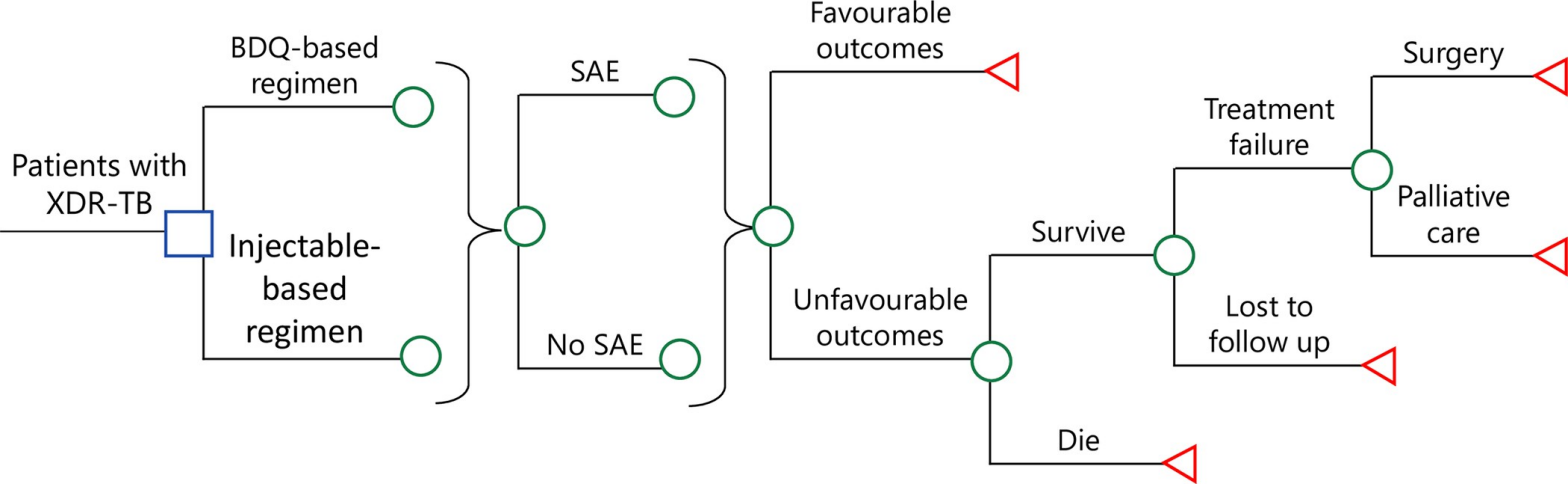
Simplified decision-analytical model for XDR-TB treatment with BDQ-based regimen versus injectable-based conventional regimen. BDQ: Bedaquiline; SAE: Serious adverse event; XDR-TB: Extensively drug-resistant tuberculosis.

A decision-analytic model (**Fig 1**) was designed to evaluate treatment outcomes of a hypothetical cohort of adult patients with active XDR-TB on two treatment options: (1) BDQ-based regimen (BDQ group), and (2) injectable-based conventional regimen (conventional group). The model time horizon was 2 years, to be consistent with the recommended 24-month treatment duration of XDR-TB [3,16]. The primary model outputs were TB-related direct medical cost and disability-adjusted life-years (DALYs).

In both study arms, all XDR-TB patients received the background 24-month treatment regimen [7]. In the BDQ group, the BDQ-based regimen consisted of 6-month intensive phase of 7 drugs (bedaquiline, linezolid, delamanid, clofazimine, terizidone, pyrazinamide, isoniazid high dose or ethionamide), followed by continuous phase with 5 drugs (linezolid, clofazimine, terizidone, pyrazinamide, isoniazid high dose or ethionamide). The BDQ treatment course included 400 mg once daily during the first 2 weeks followed by 200 mg three times per week for 22 weeks [3,16]. In the conventional group, the injectable-based regimen for XDR-TB consisted of a 6–month intensive phase of 7 drugs (capreomycin (injectable), moxifloxacin, ethionamide, terizidone, pyrazinamide, p-aminosalicylic acid, clofazimine) followed by continuation phase with 5 drugs (moxifloxacin, ethionamide, terizidone or cycloserine, pyrazinamide, p-aminosalicylic acid or clofazimine) [17]. If a serious adverse event (SAE) of the anti-TB regimen occurred, the patient would receive inpatient management. The SAE in both arms was defined as a life-threatening event resulting in hospitalization, prolongation of hospitalization, (persistent or significant) disability, or death [9,13]. By the end of the 2-year period, the patient might achieve favourable outcomes. Those who did not achieve favourable outcomes might experience treatment failure, lost follow up or death. Cases of treatment failure might undergo surgery or receive palliative care.

The model inputs (clinical, cost, and utility) were derived from published literature and public data. We did not use any global or regional dataset that requires permission to access the data. No individual patient’s data was obtained in the study, and therefore ethical approval was not required. Sources of the all model inputs are described below.

### Clinical inputs

All model inputs are shown in **Table 1**. A literature search was performed in the Medline covering the period 2000–2022 using keywords such as ‘extensively drug-resistant tuberculosis’; ‘treatment outcome’; ‘mortality’; ‘bedaquiline’; ‘injectables’; ‘standard regimen’; ‘novel drugs’, and ‘hospitalization’. The inclusion criteria for clinical studies were: (1) Reports written in English; (2) adult patients with XDR-TB; and (3) treatment outcomes and/or serious adverse events were reported. A study was included if the data relevant to the model inputs were available. The weighted average was adopted as the base-case value if multiple sources were obtained for model input, whilst the high and low values formed as the range for sensitivity analysis.

**Table 1:** Model input parameters.

Parameters	Base case value	Range for sensitivity analysis	Distribution	Reference
*Clinical inputs*				
Favourable outcome rate of injectable-based regimen	13.24%	10.59%-15.89%	Beta	[7]
Risk ratio of favourable outcome with BDQ-based versus injectable-based regimen	5.02	4.02–6.02	Lognormal	[7]
Injectable-based regimen				[7]
Mortality rate among XDR-TB patients with unfavourable outcomes	39.43%	31.54%-47.32%	Beta	
Treatment failure rate among survived XDR-TB patients with unfavourable outcomes	50.00%	40.00%-60.00%	Beta	
BDQ-based regimen				[7]
Mortality rate among XDR-TB patients with unfavourable outcomes	43.48%	34.78%-52.18%	Beta	
Treatment failure rate among survived XDR-TB patients with unfavourable outcomes	30.77%	24.62%-36.92%	Beta	
Proportion of patients who underwent surgery among treatment failure patients	30.00%	24.00%-36.00%	Beta	[18]
Incidence of SAE				
Injectable-based regimen	23.48%	18.78%-28.18%	Beta	[9]
BDQ-based regimen	7.83%	6.26%-9.40%	Beta	[13]
*Utility inputs*				
Disutility				
Active XDR-TB	0.333	0.224–0.454	Uniform	[19]
Surgery	0.490	0.392–0.588	Uniform	[20]
Palliative care	0.660	0.528–0.792	Uniform	[21]
Lost to follow-up	0.660	0.528–0.792	Uniform	Assumption
Utility decrement due to hospitalization	0.121	0.061–0.182	Uniform	[22]
Age-specific utility				[23]
18–65 years	0.92	—		
>65 years	0.84	—		
XDR-TB patient age (years)	34	18–73	Triangular	[7]
*Cost inputs* (USD)				
Drug (cost per treatment course)*				[24]
Injectable-based regimen	5,090	4,072–6,108	Gamma	
BDQ-based regimen	6,402	5,122–7,682	Gamma	
TB outpatient clinic visit (cost per visit)	31	25–37	Gamma	[5]
Number of outpatient clinic visits	25	—		[25]
Laboratory monitoring (cost per treatment course)				[5]
Injectable-based regimen	1,304	1,043–1,565	Gamma	
BDQ-based regimen	1,399	1,119–1,679	Gamma	
TB-related hospitalization (cost per day)	154	123–185	Gamma	[26]
Length of hospitalization for SAE management (days)	15	12–18	Triangular	[3]
SAE management (cost per case)				[27]
Injectable-based regimen	1,273	1018–1,528	Gamma	
BDQ-based regimen	1,160	928–1,392	Gamma	
Palliative inpatient care (cost per day)	126	48–285	Gamma	[28]
Duration of palliative care (days)	19	15–23	Triangular	[29]
Surgery (cost per case)	7,923	6,338–9,508	Gamma	[5]

BDQ: Bedaquiline; SAE: Serious adverse event; TB: Tuberculosis; XDR-TB: Extensively drug-resistant tuberculosis.

*Drugs and dosages of BDQ-based regimen and injectable-based regimen are provided in supplementary S1 Table.

A prospective clinical study reported the long-term treatment outcomes of XDR-TB patients (n = 272) who were treated with BDQ-based regimens and conventional injectable-based regimens in South Africa, and the findings were adopted as clinical model inputs. The 24-month favourable outcome rate was 13.24% in the injectable-based treatment group, and the risk ratio of favourable outcome associated with BDQ-based versus injectable-based regimens was estimated to be 5.02. The mortality rate in patients who experienced unfavourable outcomes (treatment failure, death, and lost to follow up) were 39.43% in the injectable group and 43.48% in the BDQ group. Of the survived patients with unfavourable outcomes, treatment failure rates were 50.00% and 30.77% in the injectable and BDQ-based treatment groups, respectively [7]. The proportion of patients who underwent surgery among those experienced treatment failure (30.00%) was approximated from the findings of a retrospective outcome study on a cohort of XDR-TB patients (n = 195) in South Africa [18]. The incidences of SAE with injectable-based regimen (23.48%) and BDQ-based regimen (7.83%) were retrieved from results of observational studies on drug-associated adverse events in patients with drug-resistant TB [9,13].

### Health utility inputs

The expected DALYs in each study group was estimated using the patient-time spent in a health state and the corresponding utility reduction (from the age-specific utility of healthy adults) [30]. The age-specific utility values for healthy individuals were previously generated by a health-related quality of life study on medical conditions using population measures [23]. A global burden of disease study reported the disability of active XDR-TB state to weight 0.333 [19]. The disutility of surgery (0.490) was estimated from the findings of health-related quality of life measures in 222 TB patients [20]. The disutility of palliative care (0.660) was adopted from the model input of a health economic analysis on treatment of active TB [21]. The disutility of the “lost to follow-up” state was assumed to be similar to the disutility weight for palliative care. The utility decrement of TB-related hospitalization (0.121) was adopted from the model input of a cost-effectiveness analysis of preventive measures for healthcare-associated infection [22]. DALYs resulted from XDR-TB-related mortality was approximated by the age-specific health utilities and age-specific remaining life expectancy (from the South African life tables) [31]. The base-case value of the XDR-TB patient’s age (34 years) was retrieved from the mean age of patients in the prospective clinical study on the long-term treatment outcomes of XDR-TB patients (n = 272) in South Africa [7]. The DALYs were discounted to current year with an annual rate of 3%.

### Cost inputs

The direct medical cost analysis included costs of drug acquisition, treatment monitoring, serious adverse event management, hospitalization, outpatient care, palliative care, and surgical intervention. The costs of anti-TB agents were obtained from the drug acquisition catalog of the WHO [24]. The drugs and dosages of the BDQ-based regimen and injectable-based regimen are listed in **S1 Table**. The cost of outpatient clinic visit, laboratory monitoring, and surgery were reported by a cost-analysis of diagnosis and management of drug-resistant TB in South Africa [5]. The frequency of monitoring and clinic visits adopted the recommendations of WHO and South African national TB program [3,16]. The costs of SAE management were approximated from the findings of a direct cost analysis of adverse events during treatment of multidrug-resistant TB in South Africa [27]. The cost of palliative care per day was sourced from a review of the Hospice Palliative Care Association projects in South Africa [28], and the median duration of palliative care was reported by a systematic review and meta-analysis [29]. All costs were adjusted to year 2022 using the South African consumer price index for health [32]. The costs were presented as US dollar (USD) and the currency conversion rate from South African Rand (ZAR) to USD was USD1 = ZAR14.504 [33].

### Cost-effectiveness analysis and sensitivity analysis

The analysis was performed using TreeAge Pro 2022 (TreeAge Software Inc, Williamstown, MA, USA) and Excel 365 (Microsoft Corporation, Redmond, WA, USA). If a treatment option resulted in lower DALYs at higher cost than another treatment regimen, incremental cost per DALY averted (ICER) of the more effective treatment was calculated: ICER = ΔCost/ΔDALYs.

A treatment option was accepted as the preferred cost-effective treatment if it resulted in (1) lower DALYs at lower cost, or (2) lower DALYs at higher cost and the ICER was less than the willingness-to-pay (WTP) threshold. According to the WHO recommendations, a healthcare intervention with ICER less than the gross domestic product (GDP) per capita can be considered to be highly cost-effective [34]. The GDP per capita of South Africa in 2020 was USD5,656 [35], and 5,656 USD/DALY averted was adopted as the WTP threshold in the present analysis.

All model inputs were examined by one-way sensitivity analysis over the ranges specified in **Table 1**. The probabilistic sensitivity analysis was performed using Monte Carlo simulation to examine the impact of uncertainty in all variables simultaneously. The cost and DALYs of each study arm were recalculated 10,000 times by randomly drawing each model input value from the parameter-specific distribution. The probabilities of the two study groups to be accepted as cost-effective were examined over a wide range of WTP threshold from 0 to 11,312 (2× GDP per capita) USD/DALY averted in the acceptability curves.

## Results

### Base-case analysis

The expected direct cost and DALYs of each study group in year 1 and year 2 are shown in **Table 2**. When compared to the conventional group, the BDQ group reduced 4.4152 DALYs by an incremental cost of USD1,606 (ICER 364 USD/DALY averted) over the 2-year timeframe. The ICER (364 USD/DALY averted) was lower than the WTP threshold of 5,656 USD/DALY averted, and the BDQ-based regimen was therefore the preferred cost-effective option.

**Table 2:** Results of base-case analysis.

Treatment strategy	direct cost (USD)	Incremental cost (USD)	DALYs	DALY averted
Year 1				
Conventional group	6,075	-	0.2263	-
BDQ group	6,521	446	0.2202	0.0061
Year 2				
Conventional group	408	-	7.8114	-
BDQ group	1,568	1,160	3.4023	4.4091
Total				
Conventional group	6,483	-	8.0377	-
BDQ group	8,089	1,606	3.6225	4.4152

BDQ: Bedaquiline; DALY: Disability-adjusted life-year; ICER: Incremental cost-effectiveness ratio; XDR-TB: Extensively drug-resistant tuberculosis.

### Sensitivity analysis

**Fig 2:**
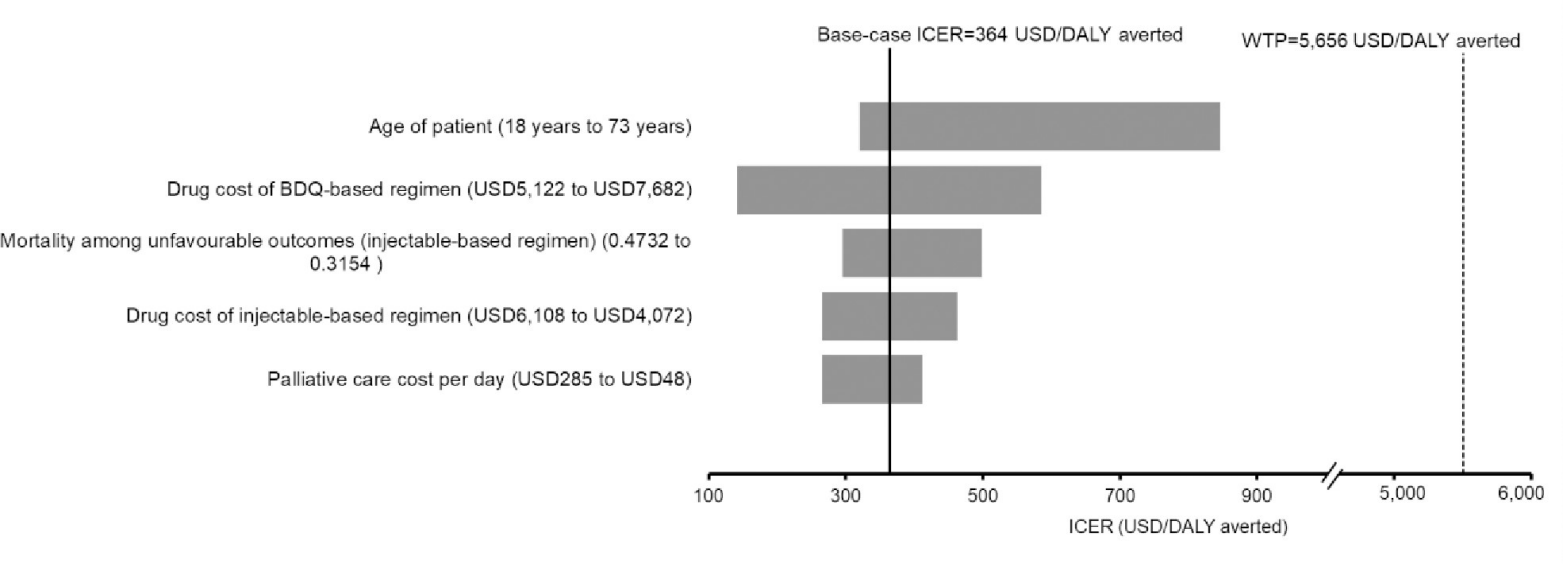
Tornado diagram of influential factors identified in one-way sensitivity analysis on ICER of the BDQ group versus the conventional group. BDQ: Bedaquiline; DALY: Disability adjusted life year; ICER: Incremental cost per DALY averted; XDR-TB: Extensively drug-resistant tuberculosis.

One-way sensitivity analysis found that the BDQ group remained to reduce DALYs at higher cost (when compared with the conventional group), and the ICER of BDQ was lower than the WTP threshold throughout variation of all model inputs over the specified ranges. The five most influential factors on the ICER of the BDQ group are shown in **Fig 2**.

**Fig 3:**
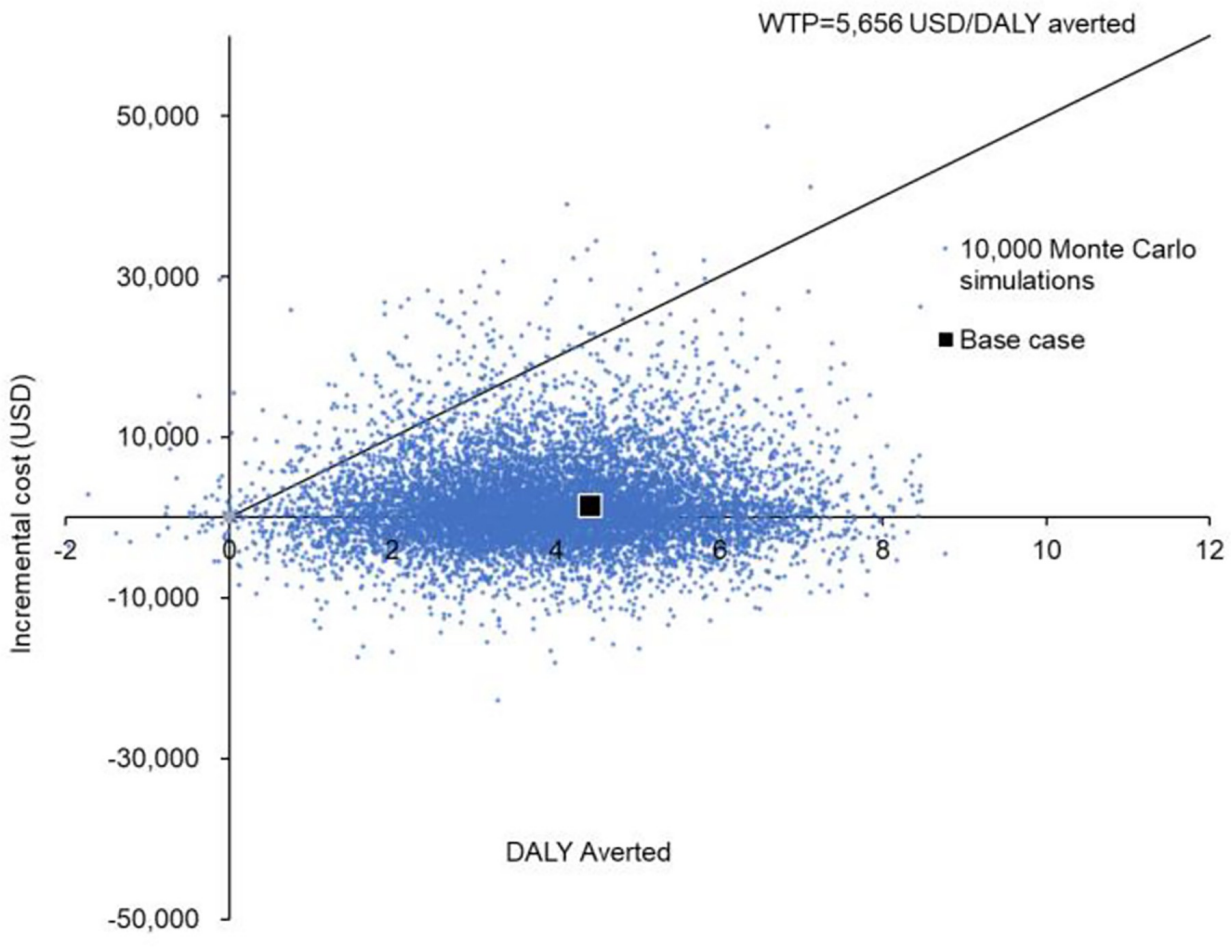
Scatter plot of the incremental cost against DALY averted by the BDQ group versus the conventional group in 10,000 Monte-Carlo simulations. BDQ: Bedaquiline; DALY: Disability-adjusted life-year; WTP: Willingness-to-pay.

Probabilistic sensitivity analysis was performed by recalculating the cost and DALYs 10,000 times with Monte Carlo simulation. The incremental cost against DALY averted by the BDQ group versus the conventional group are shown in a scatter plot (**Fig 3**). Compared with the conventional group, the BDQ group reduced DALY by 3.9162 (95%CI 3.8870–3.9454; *p*<0.001), with an incremental cost of USD1,603 (95%CI USD1,494 -USD1,712; *p*<0.001). The BDQ group reduced DALYs at lower cost in 42.68% of the time, and averted DALYs at higher cost with ICER<WTP threshold in 55.14% of the simulations. The BDQ group was therefore accepted as the preferred cost-effective option in 97.82% of the 10,000 simulations.

**Fig 4:**
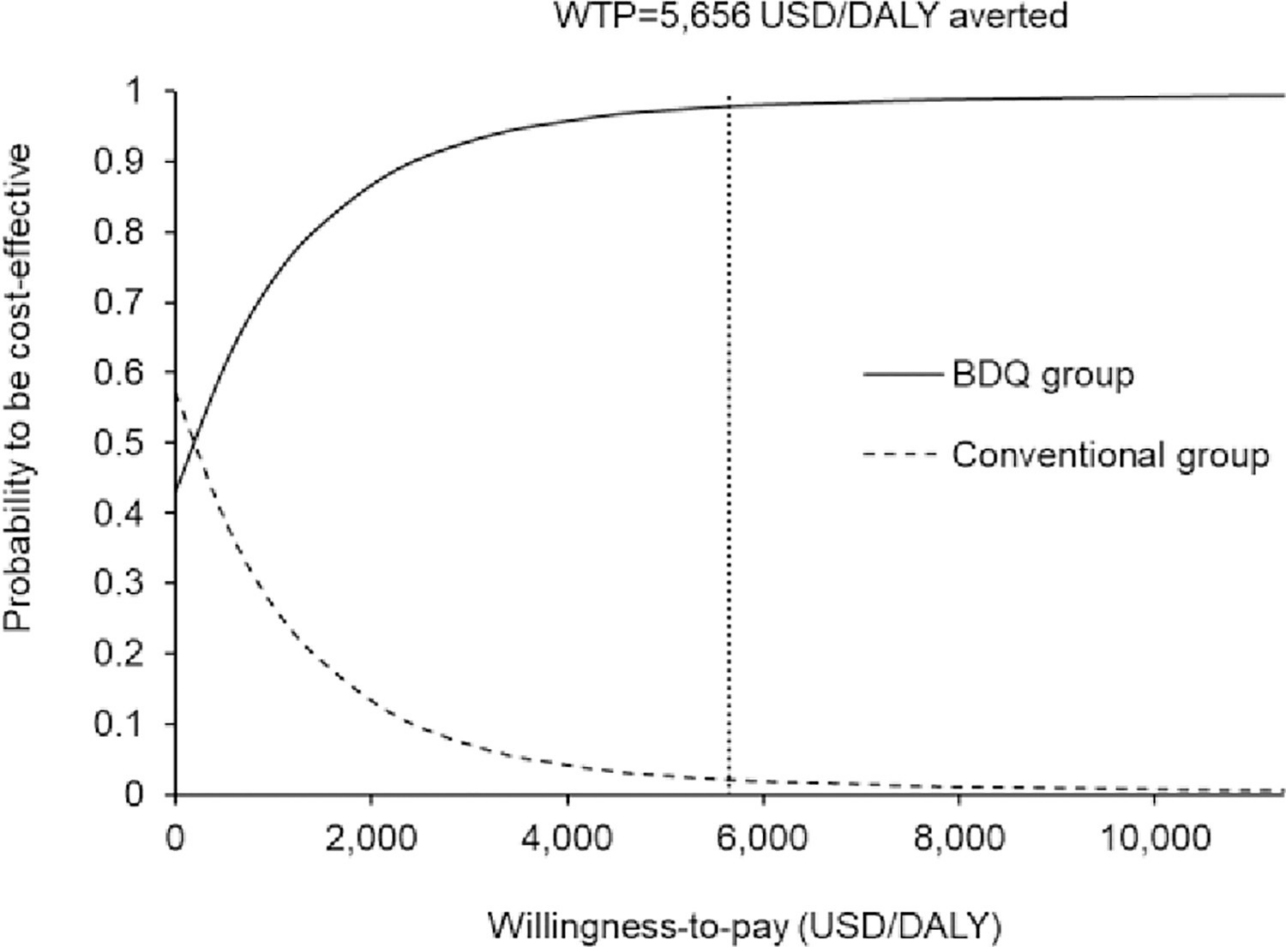
Acceptability curves of the BDQ group and the conventional group to be cost-effective against willingness-to-pay. BDQ: Bedaquiline; WTP: Willingness-to-pay.

The probabilities of the two study groups to be accepted as cost-effective are presented in the acceptability curves over a wide range of WTP (0–11,312 USD/DALY averted) (**Fig 4**). The probability to be cost-effective in the BDQ group was higher than the conventional group when the WTP was >550 USD/DALY averted, and it was >90% when the WTP threshold was >3,000 USD/DALY averted.

## Discussion

The present cost-effectiveness analysis evaluated the impact of BDQ-based regimen on the treatment outcomes of XDR-TB, measured as direct medical cost and DALYs, in a high TB burden country. The base-case analysis findings showed the BDQ-based regimen to reduce DALYs (by 4.4152 per patient) with incremental cost (by USD1,606 per patient) in patients newly diagnosed of active XDR-TB from the perspective of the South Africa national health service over a two-year timeframe. The DALY reduction in the BDQ group demonstrated in the present analysis was translated from the significantly (5-fold) improved 24-month favourable outcome rate (66.2%) associated with the BDQ-based treatment for XDR-TB versus the conventional treatment (13.24%) [7]. The incremental cost of BDQ-based regimen for XDR-TB treatment was primarily due to the higher drug acquisition cost of the BDQ-based regimen. The one-way sensitivity analysis results found the base-case results on cost, effectiveness and cost-effectiveness of the BDQ-based regimen to be highly robust, and no threshold value was identified throughout variation of all model inputs. The results of the probabilistic sensitivity analysis also supported the BDQ-based regimen to be cost-effective in over 97% of 10,000 Monte Carlo simulations at WTP of 1× GDP per capita in South Africa.

The prior health economics analyses of the BDQ-based regimen in high-burden countries were mostly focused on the treatment of multidrug-resistant TB [36–39]. Three analyses were conducted in South Africa [36–38] and one study was a multi-country analysis [39]. The multi-country analysis reported that the BDQ-based regimen was cost-saving for treatment of multidrug-resistant TB (BDQ drug cost was not included as a model input). The BDQ-based regimen was estimated to save USD43 million in the healthcare costs (versus standard regimen) by a large reduction in the number of patients who advanced from multidrug-resistant TB to XDR-TB due to initial therapy failure [39]. In the South Africa health economic analyses, the ICERs of BDQ-based regimen for multidrug-resistant TB ranged between USD516 to USD1,242 per DALY averted [37,38]. One of the three South Africa analysis found BDQ-based regimen to generate a cost saving of USD982 per DALY averted [36]. The total cost of BDQ-based regimen became lower than the conventional treatment group when the cost of treatment-related toxicity was included in the analysis [37]. One study further reported a subgroup analysis on patients who failed multidrug-resistant TB treatment (and required treatment for XDR-TB) and showed the BDQ-based regimen (versus capreomycin-based regimen) to save cost and reduce DALYs [38]. Our study showed base-case results similar to these analyses, and the findings of sensitivity analyses further supported the cost-effective robustness of the BDQ-based treatment for active XDR-TB.

All three prior analyses in South Africa were performed over a long (10 years) time horizon to capture the downstream cost and DALYs of multidrug-resistant TB-related treatment failure, lost to follow up and death [36–38]. The long-term cumulative cost avoidance resulted from improved treatment success rate had off-set the high drug cost of BDQ-based treatment and demonstrated acceptable cost-effectiveness of BDQ-based treatment for multidrug-resistant TB. Despite a short timeframe (2 years) applied in the present study, the cost avoidance and DALY averted by the BDQ-based regimen (versus conventional treatment) were adequate to demonstrate acceptable cost-effectiveness for the treatment of XDR-TB. Such prominent improvement in the cost-effectiveness of BDQ-based regimen was generated from the relatively large (5-fold) improvement in favourable outcome rate for XDR-TB by the BDQ-based treatment (66.2%) versus conventional treatment (13.24%) [8]. The relative clinical improvement of multidrug-resistant TB treatment associated with BDQ was narrower, as indicated by the 24-month cumulative probabilities of sputum conversion with BDQ-based regimen (75%) and injectable-based regimen (65%) [36].

The strength of present study included adopting the findings of a large prospective clinical study on long-term BDQ-based regimen treatment outcomes of XDR-TB patients in South Africa [7]. Our decision model also incorporated key health states of active XDR-TB (favourable outcome, SAE, treatment failure, lost to follow up and death) for evaluation of the cost and DALYs of the BDQ-based regimen versus the conventional injectable-based regimen. The decision-analytic model yet simplified the XDR-TB outcomes only to the key clinical events. The present cost analysis was conducted on direct medical costs from the perspective of healthcare provider, and indirect (such as loss of productivity) costs were not considered. The cost-effectiveness results might therefore underestimate the health economic benefits generated by the improved clinical outcomes. The base-case results were subject to the uncertainty of model inputs. All model parameters were examined vigorously in the sensitivity analyses and the base-case findings were found to be robust.

## Conclusion

The BDQ-based therapy appeared to be cost-effective, with an ICER below the WTP, and showed a high probability to be accepted as the preferred cost-effective option for active XDR-TB treatment from the perspective of national health service provider of South Africa.

## Supporting information

S1 Table The drugs and dosages of the BDQ-based regimen and injectable-based regimen for the XDR-TB treatment. BDQ: Bedaquiline; XDR-TB: Extensively drug resistant tuberculosis.
(DOCX)
