# Supplementary material for: Bedaquiline-based treatment for extensively drug-resistant tuberculosis in South Africa: A cost-effectiveness analysis
Source: PLoS One. 2022 Aug 5;17(8):e0272770. doi: 10.1371/journal.pone.0272770 (PMC9355220; doi:10.1371/journal.pone.0272770)
Supplement: S1 Table — BDQ: Bedaquiline; XDR-TB: Extensively drug resistant tuberculosis. (DOCX) [file pone.0272770.s001.docx]

**Table S1** The drugs and dosages of the BDQ-based regimen and injectable-based regimen for the XDR-TB treatment. BDQ: Bedaquiline; XDR-TB: Extensively drug resistant tuberculosis

| Regimen | Phases of treatment and drugs | Regimen |
| --- | --- | --- |
| BDQ-based regimen | Bedaquiline | 400 mg orally once daily for 2 weeks followed by 200 mg 3 times per week for 22 weeks |
|  | Linezolid | 600mg orally daily (24 months) |
|  | Delamanid | 100 mg orally twice daily (24 weeks) |
|  | Clofazimine | 100-300mg orally daily (24 months) |
|  | Terizidone | 10-15 mg/kg/day orally, maximum 1000 mg/day once daily (24 months) |
|  | Pyrazinamide | 25 mg/kg (20-30 mg/kg) orally once daily (24 months) |
|  | Isoniazid high dose | 10-15mg/kg orally once daily (24 months) |
|  | Ethionamide | 15 to 20 mg/kg/day, orally once daily (maximum daily dose of 1 gram) (24 months) |
| Conventional injectable-based regimen | Capreomycin (injectable) | 1 g daily (not to exceed 20 mg/kg/day) given intramuscularly or intravenously (intensive phase of 24 weeks) |
|  | Moxifloxacin | 400mg orally once daily (24 weeks of intensive phase) |
|  | Ethionamide | 15 to 20 mg/kg/day, orally once daily (maximum daily dosage of 1 gram) (24 months) |
|  | Terizidone | 10-15 mg/kg/day orally, maximum 1000 mg/day once daily (24 months) |
|  | Pyrazinamide | 25 mg/kg (20-30 mg/kg) orally once daily (24 months) |
|  | Para-aminosalicylic acid (PAS) | 8–12 g/day orally in 2–3 divided doses (24 months) |
|  | Clofazimine | 100-300mg orally daily (24 months) |

The drug cost estimation was based upon the body weight of 52 kg [1].

Reference:

1. Olayanju O, Limberis J, Esmail A, Oelofse S, Gina P, Pietersen E, et al. Long-term bedaquiline-related treatment outcomes in patients with extensively drug-resistant tuberculosis from South Africa. European Respiratory Journal. 2018;51(5):1800544.
